# Exposure to CFTR Modulators During Pregnancy in Cystic Fibrosis: Four Cases to Highlight Neonatal Diagnostic Challenges and Outcomes

**DOI:** 10.3390/ijns12010011

**Published:** 2026-02-26

**Authors:** Louis Domenach, Adrien Pagin, Camille Cisterne, Marie-Pierre Audrezet, Laure Couderc, Laetitia Monteil, Léa Roditis, Marlène Murris, Julie Macey, Michael Fayon, Stéphanie Bui, Marie-Pierre Reboul

**Affiliations:** 1 Laboratoire de Génétique Moléculaire, CHU de Bordeaux, 33076 Bordeaux Cedex, France; marie-pierre.reboul@chu-bordeaux.fr; 2 Service de Toxicologie et Génopathies, CHU de Lille, 59037 Lille, France; adrien.pagin@chu-lille.fr; 3 Service de Pneumologie-Allergologie Pédiatrique, CHU de Lille, 59037 Lille, France; camille.cisterne@chu-lille.fr; 4 Laboratoire de Génétique Moléculaire, CHU de Brest, 29609 Brest, France; marie-pierre.audrezet@chu-brest.fr; 5 CRCM Pédiatrique, Département de Pédiatrie Médicale, CHU de Rouen, 76031 Rouen, France; laure.couderc@chu-rouen.fr; 6 Service de Génétique Médicale, CHU de Toulouse, 31300 Toulouse, France; monteil.l@chu-toulouse.fr; 7 CRCM Pédiatrique, Service de Pneumo-Allergologie Pédiatrique, CHU de Toulouse, 31059 Toulouse, France; roditis.l@chu-toulouse.fr; 8 CRCM Adulte, Service de Pneumologie-Allergologie, CHU de Toulouse, 31059 Toulouse, France; murris.m@chu-toulouse.fr; 9 CRCM Adulte, Service de Pneumologie, CHU de Bordeaux, 33600 Pessac, France; julie.macey@chu-bordeaux.fr; 10 Département de Pédiatrie, CIC-P INSERM 1401, Centre de Recherche Cardio-Thoracique de Bordeaux, INSERM U1045, CHU Bordeaux & Université de Bordeaux, 33000 Bordeaux, France; michael.fayon@chu-bordeaux.fr (M.F.); stephanie.bui@chu-bordeaux.fr (S.B.)

**Keywords:** CFTR, cystic fibrosis, newborn screening, pregnancies, CFTR modulators, case reports

## Abstract

CFTR modulators have transformed the clinical evolution of patients with CF. The number of pregnancies is increasing in women with CF, most of whom are now treated with CFTR modulators such as elexacaftor/tezacaftor/ivacaftor (ETI) or Tezacaftor/Ivacaftor. This raises some questions as we still lack data on foetal and maternal safety. The preliminary data seem to support the continuation of modulators. Some of these mothers may also give birth to newborns with CF and this raises more questions. We report here four cases of CF newborns whose mothers were treated with CFTR modulators throughout pregnancy to help refine potential foetal outcomes of in utero administration of CF modulators. No maternal or foetal complications could be attributed to CFTR modulators. Three CF newborns were exposed to ETI and were false negative of the newborn screening. Two of them were pancreatic sufficient at birth. The remaining patient, exposed to Tezacaftor/Ivacaftor (TI) alone, showed elevated immunoreactive trypsin (IRT) and severe pancreatic insufficiency at birth. These cases highlight that in utero administration of ETI could potentially improve neonatal outcomes of CF newborns and cause newborn screening false negative.

## 1. Introduction

The advent of CFTR modulators has revolutionised the prognosis of cystic fibrosis (CF) in recent years, particularly with the latest combination of elexacaftor, tezacaftor and ivacaftor (ETI). The effects on lung function, quality of life and BMI are remarkable [1]. The data seem to indicate that life expectancy and transplant risk for all CF patients have improved significantly since then [2], but most of this improvement is due to modulator effects and a significant part (around 10% in Europe and US) of the patients still have no access to CFTR modulators due to unresponsive genotypes. One of these findings is the increase in pregnancies in women with cystic fibrosis [2], which raises several questions regarding the continuation of this therapy during pregnancy. Data on risks and benefits for mother and foetus are still limited but generally support the continuation of therapy. There appears to be a risk of relapses for mothers who discontinue ETI, and most complications during these pregnancies do not appear to be related to ETI [3]. The main reported risks to foetuses or children exposed to ETI appear to be ophthalmological and hepatic, with some cases of cataracts and elevated liver enzymes reported.

Most of these patients give birth to children without CF, although foetuses with CF can occur in some pregnancies. Reports and data on such pregnancies are even rarer, and only one case has been described to date [4]. The mother was homozygous for F508del and continued ETI throughout the pregnancy. She delivered a F508del homozygous infant without complications. There was no meconium ileus, no pancreatic insufficiency and no respiratory distress. An important finding was the negative newborn screening due to an IRT below the detection threshold. The diagnosis of CF was made by the genetic analysis requested by the paediatricians. In another context, two other cases of newborns with cystic fibrosis born after exposure to ETI during pregnancy were also described. In these cases, the mothers were carriers [5,6] and took ETI to treat foetal meconium ileus. It was decided to administer ETI to the healthy mother during the third term and the ileus completely resolved. One neonate had elevated immunoreactive trypsinogen (IRT) but no pancreatic insufficiency, while the other had borderline IRT and pancreatic insufficiency.

Most newborn screening programs are based on the measurement of IRT in the first week of life, followed by genetic analysis of the main variants responsible for CF and the sweat chloride test. Despite excellent sensitivity, it is not possible to completely exclude false-negative results. Some factors, such as the presence of meconium ileus or rare variants, are already well recognised. However, the use of CFTR modulators during pregnancy appears to contribute to false-negative results in newborn screening, a situation that is likely to increase in the coming years.

We present here four new cases of CF newborns whose mothers were treated with CFTR modulators during pregnancy. The pregnancies were without complications and in most of the newborns the neonatal outcome was favourable, especially in terms of digestive and pulmonary functions. We would also like to emphasise the possibility of hidden pancreatic insufficiency and negative neonatal screening at birth for most of them. We believe that these cases may provide valuable data on the effects of CFTR modulators in utero in CF newborns and highlight the diagnostic challenges they pose.

## 2. Case Reports

All clinical data were collected as part of the standard clinical procedure in the respective hospitals. The most important data are listed in Table 1. The four patients were followed in different cities.

**Patient 1:** Born to a c.1521_1523del (F508del) homozygous mother and a heterozygous father. ETI was administered to the mother shortly before conception and continued throughout the pregnancy. The couple initially opted out of prenatal diagnosis but eventually underwent it due to the increased nuchal translucency. The *CFTR* analysis revealed F508del homozygosity. The rest of the pregnancy was uneventful and the mother spontaneously gave birth to a healthy newborn at 37 WG. The mother decided not to breastfeed. Repeated ophthalmological examinations in the first year revealed no evidence of cataract. Abdominal ultrasound was unremarkable and liver function tests showed a slight increase in ASAT (<2 upper limit of N). Faecal elastase remained normal for the first 6 months. He is now 1 year and 6 months old and still exhibits no respiratory symptoms. The presence of vas deferens has not been assessed. Growth and psychomotor development are presently normal. He started Lumacaftor/Ivacaftor at one year of age as he was homozygous for F508del. ETI will be started as soon as he is eligible, when he reaches 2 years old.

**Patient 2:** Born to a c.1521_1523del (F508del) homozygous mother and a father heterozygous for a large deletion of *CFTR* encompassing exons 19 to 21, c.2988+1173_3468+2111del. The mother had an ectopic pregnancy that was medically terminated 3 months after the onset of ETI and 1 month prior to this pregnancy. The couple decided not to undergo prenatal *CFTR* analysis. ETI was continued throughout the pregnancy. The patient was delivered by caesarean section at 34 + 2 WG as the mother’s general condition deteriorated. Neonatal screening was negative for CF and the patient was discharged from hospital on day 5. However, on day 21, he was admitted to the intensive care unit due to apnoea episodes and supraventricular tachycardia. A *CFTR* analysis was performed, which revealed compound heterozygosity for F508del and the paternal deletion. In the first months of life, he suffered from severe gastro-oesophageal reflux and digestive problems. He was breastfed until 2 months of age before mixed feeding with formula. The sweat test was intermediate at 1.5 months (32 mmol/L) and positive at 8 months (104 mmol/L). He is now 2 years old, the ophthalmological examination shows no signs of cataract and ETI was introduced recently as his first CFTR modulator treatment. The presence of vas deferens has not been assessed. Psychomotor development is marked by poor language skills and aggressiveness.

**Patient 3:** Born to a c.1521_1523del (F508del)/c.3454G>C (D1152H) compound heterozygous mother and a c.496A>T (K166*) heterozygous father. The mother had already had a first pregnancy and gave birth to a healthy girl who was not a carrier of the paternal variant. She started TI in association with ivacaftor after the first pregnancy. After TI dose reduction and ivacaftor discontinuation due to mood and sleep disorders, she became pregnant a second time. Non-invasive prenatal testing revealed the presence of the paternal variant. No invasive prenatal diagnosis was performed. As the maternal allele transmitted to the foetus was not known and the mother was stable with TI, she decided (after multidisciplinary discussions with CF team) to continue TI throughout the pregnancy without discontinuation or switch to ETI. There were no complications for her or the foetus. She gave birth to a healthy newborn with no neonatal complications apart from liquid faeces. The newborn screening test was positive with an elevated IRT (125 µg/L), and faecal elastase was decreased (<15 µg/g) on day 7. At 1 month he had bronchiolitis and sweat chloride test was elevated at 2.5 months (81 mmol/L). Molecular analysis revealed the presence of the F508del maternal allele and the K166* paternal allele. He is now 4 months old and the ophthalmological examination showed no signs of cataract. Neurodevelopment is not impaired. The presence of vas deferens has not been assessed. He is not treated with any modulators as he is not eligible for one now. ETI should be started when he reaches 2 years old.

**Patient 4:** Born to a c.1521_1523del (F508del) homozygous mother and a c.1196C>A (A399N) heterozygous father. The mother had been treated with ETI throughout the whole pregnancy. Prenatal diagnosis was performed by amniocentesis after non-invasive prenatal diagnosis showed the presence of the paternal allele, resulting in foetal compound heterozygosity for F508del and A399N. Foetal amniotic fluid digestive enzymes were measured to help evaluate the potential pathogenicity of the A399N variant and were normal for the term. The A399N was classified as a variant of uncertain significance (VUS) and CF could not be confirmed. No digestive obstruction and no complications occurred during pregnancy or birth. The newborn had a negative neonatal screening (IRT 26 µg/L). The sweat chloride test was positive, in favour of a CF and a potential pathogenicity of A399N variant. The neonate remained pancreatic sufficient until 6 months of age and began to show pulmonary symptoms, including polypnoea, with a pulmonary consolidation noted on chest X-ray. The presence of vas deferens has not been assessed.

## 3. Discussion and Conclusions

We present here four cases of CF newborns exposed to CFTR modulators in utero. In all cases, the mothers were treated with modulators prior to pregnancy and chose to continue their use.

**Concerning maternal health and foetal risks:** Although recommendations for the use of CFTR modulators during pregnancy are gradually emerging [7,8], this decision must be carefully considered by both the mother and the healthcare team. The mother should be fully informed of the potential risks to herself and her foetus and of the need for close monitoring of the newborn, including ophthalmological examinations and liver tests. Although the initial data appear reassuring [3], there is still a lack of long-term follow-up for these children. The four cases we present here confirm these preliminary results, as there were no complications during pregnancy or delivery that could be attributed to CFTR modulators, none of the newborns showed signs of cataract, and only patient 1 had a slight increase in liver enzymes. These patients are still young, so the possibility of long-term toxicity from in utero exposure to ETI cannot be ruled out. The speech delay and behavioural abnormalities reported in patient 2 cannot be directly attributed to ETI side effects, as many other factors such as genetics or environment could explain them. However, psychiatric disorders appear to be possible complications of ETI administration [9]. Although they tend to be reported in older age groups [10], the causality remains unclear in this case. Such signs should be detected early, treated and monitored closely in young people exposed to ETI.

**Concerning laboratory tests and newborn screening:** One interesting finding in our cases was the pancreatic function. Exocrine pancreatic insufficiency is a usual finding for most CF patients, especially those carrying F508del, and more than half of CF newborns present with pancreatic insufficiency at birth [11]. Most CF patients require an enriched diet, often supplemented with pancreatic enzymes, fat-soluble vitamins, or NaCl. In this case, patient 1 was fully pancreatic sufficient at birth and only began to show biological signs of insufficiency weeks after birth. He also had normal IRT levels, which is a pancreatic proenzyme and a highly effective tool for screening CF newborns. These findings are similar to the first report of a CF newborn exposed to ETI throughout the entire pregnancy [4], except that our patient became pancreatic insufficient a few months after birth. As ETI may pass through milk [12], the fact that the mother of patient 1 did not breastfeed may help explain why the pancreatic function did not remain sustained for longer. Interestingly, patient 2 also had normal IRT and intermediate sweat chloride test at 1.5 months, but faecal elastase appeared lower, even though it was not measured at the exact same age. Patient 3 had a positive newborn screening test and a much more severe phenotype with complete pancreatic insufficiency at birth. He also carried one variant predicted to be unresponsive to modulators and was not exposed to ETI but Tezacaftor/Ivacaftor. The impact of ETI on gastro-intestinal outcomes is still unclear, but it does seem that early administration could benefit some patients, especially younger ones, as ETI may restore pancreatic function when administered early [13]. The two reports of in utero treatment of meconium ileus with ETI [4,5] could help reinforce that idea. Finally, patient 4 brings a new finding as foetal digestive enzymes were normal and the newborn still presented with a positive sweat test. This is something that we have never encountered in our experience. Foetal digestive enzyme measurement is usually a useful test to help detect CF in foetuses with variants of uncertain significance, offering high sensitivity [14]. The A399N is still considered as a VUS and the very mild clinical presentation of patient 4 could either be due to a CF differential diagnosis or a potential effect of in utero administration of ETI. This case may suggest that foetal digestive enzymes would be less suitable for VUS assessment in foetuses exposed to ETI in utero and should warrant further explorations.

**Concerning shared decision-making and multidisciplinary approach:** These situations highlight the necessity of multidisciplinary discussions involving a CF expert team together with obstetricians, geneticists, paediatricians, and all other healthcare professionals involved, as well as the active participation of patients. Decisions should be made in the context of multidisciplinary team meetings (MDT) with the patients remaining at the centre of the decision-making process. Anticipating prenatal testing, neonatal screening challenges, and postnatal management is essential if modulators are maintained during pregnancy particularly when the foetus is affected by CF. More data are being gathered, and expert opinions, workshops or recommendations start to emerge [15,16]. Current evidence suggests that the benefit/risk of in utero ETI administration is favourable. There is potential efficacy in preventing neonatal complications in foetuses with CF and with limited risk for both the mother and the foetus. The benefits are maybe even greater when the mother has CF herself and is already taking ETI as discontinuation of modulators may have negative outcomes for the mother. All these data must be confirmed with prospective studies to fully comprehend them and help the decision-making process for CF expert team.

In summary, these cases confirm that modulators, and ETI in particular, can interfere with prenatal diagnosis and newborn screening. IRT and foetal digestive enzyme levels must be interpreted with caution, and gastrointestinal or respiratory symptoms may be mild. Continuation of the analyses and referral to a specialised centre should be considered in these situations, as this begins to appear in expert recommendations [15].

We also believe that these data could contribute to a better understanding of the potential beneficial effects of ETI in CF newborns when exposed in utero or in the first months of life. For the time being, we rely mainly on case reports to guide our practice. However, further prospective studies need to be conducted to fully understand the effects of modulators on mothers and newborns clinical outcomes and biological markers when patients were exposed in utero or a few months after birth.

## Figures and Tables

**Table 1:** **Summary of cases characteristics.** IRT threshold value at Day 3 is 60–65 µg/L, sweat chloride test threshold values are considered negative if <30 mmol, positive ≥ 60 mmol/L and intermediate between these values. Faecal elastase threshold value for pancreatic insufficiency is <200 µg/L. NA = Not Assessed.

	Patient 1	Patient 2	Patient 3	Patient 4
**Genotype**				
Maternal	F508del/F508del	F508del/F508del	F508del/D1152H	F508del/F508del
Paternal	F508del heterozygous	dele19_21 heterozygous	K166* heterozygous	A399N heterozygous
Foetal	F508del/F508del	F508del/dele19_21	F508del/K166*	F508del/A399N
**Pregnancy**				
Modulators	ETI continued during entire pregnancy	ETI continued during entire pregnancy	Tezacaftor/Ivacaftor continued during entire pregnancy	ETI continued during entire pregnancy
Maternal complications	None	None	None	NA
Foetal complications	T1 Nuchal translucency = 3 mm	None	None	None, normal amniotic fluid enzymes
Delivery	37 WG, spontaneous, vaginal no complications	34 WG + 2, Caesarean	35 WG + 3 days	NA
**Postnatal**				
Neonatal complications	None	ICU at M1 for apnea and episodes of supraventricular tachycardia	Intestinal Malabsorption with liquid stools	NA
Lungs	Minor thoracic distention, no symptoms	Obstructive ventilatory defect, right upper lobe atelectasis, respiratory symptoms	Bronchiolitis D28	Polypnea, persistent consolidation on chest x-ray
Liver	Unremarkable	Unremarkable	NA	NA
Pancreas	Sufficient until 6 months	Pancreatic insufficiency at M1	Pancreatic insufficiency	Sufficient
Eyes	No cataract	No cataract	No cataract	No cataract
Breastfeeding	No	2 months then mixed feeding	1 month then mixed feeding	NA
**Newborn Screening/Biology**				
IRT	14.7 µg/L	34.7 µg/L	125 µg/L	26 µg/L
Sweat chloride test	90 mmol/L (M3)	32 mmol/L (M1.5) 104 mmol/L (M8)	81 mmol/L (M2.5)	69 mmol/L
Faecal Elastase	427 µg/g (D3) 151 µg/g (D16) 43 µg/g (M8)	105 µg/g (M1)	<15 µg/g (D7)	NA

## Data Availability

The data supporting the findings of this study are not publicly available due to patient privacy and confidentiality restrictions. Relevant data are available from the corresponding author upon reasonable request and with appropriate ethical approvals.

## Abbreviations

The following abbreviations are used in this manuscript:

CFTR	Cystic Fibrosis Transmembrane Conductance Regulator
CF	Cystic Fibrosis
ETI	Elexacaftor/Tezacaftor/Ivacaftor
BMI	Body Mass Index
IRT	ImmunoReactive Trypsinogen
WG	Weeks of Gestation
ASATA	Aspartate AminoTransferase
